# Placental growth factor level is correlated with intrapartum fetal heart rate findings

**DOI:** 10.1186/s12884-022-04562-w

**Published:** 2022-03-17

**Authors:** Hiroaki Tanaka, Kayo Tanaka, Sho Takakura, Naosuke Enomoto, Shintaro Maki, Tomoaki Ikeda

**Affiliations:** Department of Obstetrics and Gynecology, Mie University, Tsu, Japan

**Keywords:** Biomarker, Delivery Placental growth factor, Fetal heart rate monitoring

## Abstract

**Objective:**

Here, we tested the correlation between maternal placental growth factor (PlGF) and fetal heart rate (FHR) monitoring findings.

**Methods:**

We included 35 women with single pregnancies from 35 to 42 weeks of gestation who were hospitalized owing to onset of labor. Blood samples were collected at the start of labor. Intrapartum FHR monitoring parameters included total deceleration area, average deceleration area (mean deceleration area per 10 min), and five-tier classification level.

**Results:**

Of the 35 women, 26 (74%) had vaginal delivery and 9 (26%) had cesarean section. After excluding 2 women who had cesarean section for arrest of labor, we analyzed 26 women who had vaginal delivery (VD group) and 7 who had cesarean section for fetal indications (CSF group). PlGF level was significantly higher in the VD group (157 ± 106 pg/ml) than in the CSF group (74 ± 62 pg/ml) (*P* = 0.03). There were no significant correlations between PlGF and total (*r* = -0.07) or average (*r* = -0.08) deceleration area.　There was a significant negative correlation (*r* = -0.42, *P* = 0.01) between PlGF and the percentage of level 3 or higher in the five-level classification.

**Conclusion:**

PlGF was correlated with FHR monitoring findings and might be a promising biomarker of intrapartum fetal function.

## Synopsis

PlGF measured before labor may be useful in predicting cesarean section due to fetal dysfunction. In addition, PlGF has a significant correlation with FHR findings at delivery by five-tier classification system.

## Introduction

Conversion to cesarean section can occur for various reasons, such as non-reassuring fetal status and arrest of labor. The risk factors for non-reassuring fetal status include fetal growth restriction, oligohydramnios, and post-term delivery. These risk factors, along with fetal well-being, are assessed before delivery. However, even when vaginal delivery (VD) is possible, repeated uterine contractions during delivery may lead to non-reassuring fetal status—an indication for conversion to cesarean section [1]. At present, it is difficult to predict whether VD will be successful, with no signs of non-reassuring fetal status.

Various placenta-derived molecules have been proposed as markers of fetal–placental function. These include estriol, a placental steroid metabolite [2] human placental lactogen (hPL), a hormone secreted by syncytiotrophoblasts of placental villi [3] and placental growth factor (PlGF) and soluble fms-like tyrosine kinase-1 (sFlt-1), two proteins secreted by trophoblasts of placental villi [4, 5]. Angiogenesis is an essential process in the establishment of pregnancy, and many factors, including those of the vascular endothelial growth factor (VEGF) family (such as PIGF), are intricately involved in regulating growth [1]. PIGF enhances the activity of VEGF by competitively binding to the VEGFR-1 receptor, allowing VEGF to bind to VEGFR-2, which has strong tyrosine kinase activity, thereby promoting angiogenesis [1]. PLGF is markedly increased during pregnancy. There are conflicting reports on whether PlGF contributes to the invasion of trophoblastic cells [3, 4].

In PE, sFlt-1 increases and PlGF decreases in maternal blood owing to impaired placental return caused by impaired remodeling of spiral arteries [5, 6]. This decrease in PlGF is a combination of reduced PlGF expression in the placenta due to excessive placental hypoxia and reduced free PlGF bound to elevated sFLT-1. Therefore, the ratio of sFlt-1 to PlGF is likely to be more effective than PlGF alone in predicting the development of preeclampsia (PE) [7].

In normal pregnant women, sFlt-1 increases from 32 weeks on, while PlGF decreases from around 30 weeks on [8]. This decrease in PlGF is an increase in sFlt-1; the decrease in placental function in late pregnancy that is not associated with PE is not an impairment of placental return like PE, but an impairment of diffusion in the placenta [9]. Therefore, there is no extreme increase in Flt-1 as the one that happens in PE, and an excessive increase in sFlt-1 does not cause an excessive decrease in PlGF. Conversely, since the expression of PlGF decreases in response to decreased placental oxygenation, we hypothesized that PlGF alone is decreased in placental hypofunction not associated with PE (impaired placental return in late pregnancy). For these reasons, PlGF was taken as a predictive marker of delivery outcome in this study. We tested the correlation between maternal PlGF and fetal heart rate (FHR) monitoring findings. The goal was to understand whether PlGF could serve as a predictive marker for delivery outcomes.

## Methods

### Study design and participants

This study included primiparous women at 35 to 42 weeks of gestation who were admitted to Mie University Hospital due to onset of labor from June to September 2020. Exclusion criteria were as follows: cesarean section for medical indications at the onset of labor, fetal abnormalities, gestational diabetes, preeclampsia, and fetal growth restriction. Gestational diabetes was diagnosed according to the criteria of the International Association of Diabetes and Pregnancy Study Groups Consensus Panel. Preeclampsia was diagnosed according to the criteria of the International Society for the Study of Hypertension in Pregnancy. Fetal growth restriction was diagnosed according to the criteria of the American College of Obstetricians and Gynecologists [10]. We excluded women who had cesarean section for non-fetal indications.

### Measurement of PlGF

Blood samples to measure maternal PlGF were collected at the start of labor. After the onset of labor, the participants were divided into two groups: the VD group and the Cesarean section for fetus (CSF) group. PlGF measured before the onset of labor was compared between the VD group and the CSF group. We analysis hazard ratio of the occurrence of a cesarean section in age (median > 33 years), gestational weeks (median > 37 weeks), primipara, body mass index (median > 25), PlGF (median < 100 pg/ml).

### Correlation between birth weight, pH of umbilical artery and PlGF

We measured birth weight and the pH of the umbilical artery, and examined the correlation between these values and PlGF at delivery.

### Intrapartum FHR monitoring

FHR monitoring started after the onset of labor and was recorded continuously until delivery. The onset of labor was defined as regular contractions of the uterus with pain at intervals of less than 10 min. From the intrapartum FHR monitoring records, we calculated total deceleration area, deceleration area per 10 min, and the proportion of five-tier classification level 3 or higher.

Deceleration area was measured using the method of Cahill et al. [11]. Briefly, we calculated total deceleration area as the sum of the areas within the deceleration, and each area was estimated as 1/2 × duration × depth in the final 120 min of electronic fetal monitoring as a measure of both quantity and severity. Only measurements from the first stage of labor were used. In addition, the average deceleration area per 10 min was calculated as follows: (total deceleration area / recording time) × 10.

Intrapartum FHR monitoring using the five-tier classification system [12] is described in Tables 1 and 2. FHR monitoring data were output every 10 min and evaluated according to the five-tier classification system. In this classification, level 3 or higher indicates the need to prepare for a rapid delivery. Therefore, we calculated the proportion of cases in level 3 or higher.

**Table 1:** Maternal and neonatal characteristics

Characteristic	VD group (*n* = 26)	CSF group (*n* = 7)	*P*-value
Age (years)	32.6 ± 0.7	31.6 ± 1.6	0.57
Primipara, *n* (%)	18 (69%)	6 (85%)	0.35
Height (cm)	157.1 ± 0.9	156.7 ± 1.8	0.84
Weight (kg)	63.5 ± 2.0	62.3 ± 3.9	0.79
Body mass index	25.7 ± 1.3	25.3 ± 1.7	0.81
Gestational age at birth (weeks)	38.5 ± 0.3	37.7 ± 0.6	0.24
Maternal complications, n (%)	0 (0%)	0 (0%)	-
Smoking	0 (0%)	0 (0%)	-
Birth weight (g)	2950 ± 85	2584 ± 163	0.06
pH of umbilical artery	7.28 ± 0.01	7.14 ± 0.02	0.001
Sex of newborn infant			0.87
Male	12 (46%)	3 (43%)	
Female	14 (54%)	4 (57%)	

*CSF* Cesarean section for fetal indications, *VD* Vaginal delivery

Data are the mean ± standard deviation unless otherwise indicated

**Table 2:** Five-tier classification of fetal heart rate deceleration

Fetal heart rate variability	Five-tier classification							
	No	ED	Mild VD	Severe VD	Mild LD	Severe LD	Mild PD	Severe PD
**Moderate variability**								
Normal baseline	1	2	2	3	3	3	3	4
Tachycardia	2	2	3	3	3	4	3	4
Bradycardia ≥ 80 bpm	3	3	3	4	3	4	4	4
Bradycardia < 80 bpm	4	4	-	4	4	4	-	-
**Minimal variability**								
Normal baseline	2	3	3	4	3	4	4	5
Normal baseline	3	3	4	4	4	5	4	5
Bradycardia ≥ 80 bpm	4	4	5	5	5	5	5	5
Bradycardia < 80 bpm	5	5	-	5	5	5	-	-
**Absent variability**	4	5	5	5	5	5	5	5
**Marked variability**	2	2	3	3	3	4	3	4
**Sinusoidal**	4	4	4	4	5	5	5	5

*ED* Early deceleration, *LD* Late deceleration, *PD* Prolonged deceleration, *VD* Variable deceleration

Deceleration was classified as mild or severe, with severe defined as follows and everything else mild: severe VD, the lowest point of transient bradycardia was < 70 bpm and lasted for ≥ 30 s, or the lowest point was ≥ 70 bpm and < 80 bpm, and lasted for ≥ 60 s; severe LD, the largest drop in heart rate from baseline was ≥ 15 bpm; and severe PD, the lowest point was < 80 bpm

### Assessment of serum markers

Serum samples (2 ml), collected according to a standard operating procedure, were analyzed. Maternal serum levels of PlGF were determined by means of the fully automated Elecsys assays for PlGF on an electrochemiluminescence immunoassay platform (Cobas e411 analyzers, Roche Diagnostics K. K.). The within-run coefficient of variation for control samples is below 4% for an assay. Between-run coefficients of variation are 2.3% to 5.6% for the Elecsys PlGF assay.

### Statistical analysis

All values are presented as the mean ± standard deviation. All statistical analyses were performed using GraphPad Prism 8 (GraphPad, San Diego, CA). Hazard ratio was used for Cox regression analysis. Pearson product-moment correlation coefficient analysis was used to measure the correlation between PlGF level and FHR monitoring parameters. Values of *P* < 0.05 were considered statistically significant.

## Results

### Maternal and neonatal characteristics

**Fig. 1:**
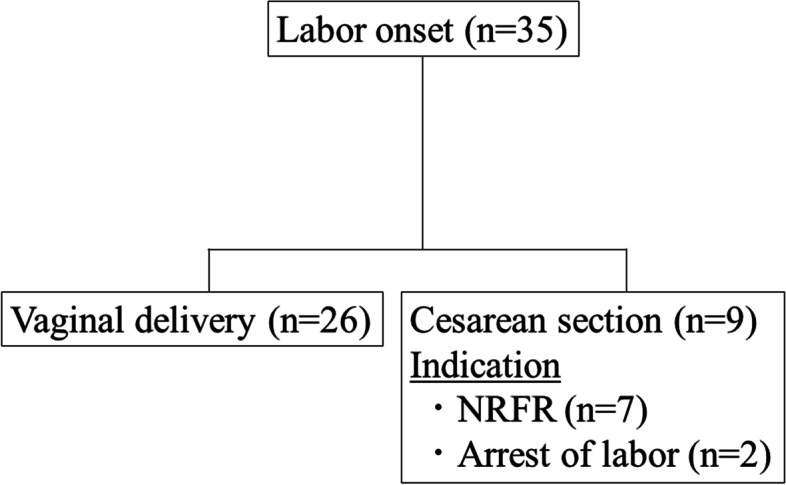
Flow diagram of patient enrollment

We included 35 women with single pregnancies from 35 to 42 weeks of gestation who were hospitalized due to onset of labor and gave their informed consent to participate in the study (Fig. 1). Of the 35 women, 26 (74%) had VD, and 9 (26%) had cesarean section; cesarean section was performed for fetal indications in 7 and arrest of labor in 2 (Fig. 1). After excluding 2 women who had cesarean section for non-fetal indications (arrest of labor), we analyzed 26 women who had VD (VD group) and 7 who had cesarean section for fetal indications (CSF group).

The maternal and neonatal characteristics of the VD and CSF groups are shown in Table 3. There were no significant differences in maternal and neonatal characteristics between the two, except umbilical artery pH, which was significantly higher in the VD group than in the CSF group (*P* = 0.001).

**Table 3:** Five-tier classification system

Level	Correspondence
1	Observation
2	Observation or Increase monitoring, implement conservative treatments, search for cause
3	Increase monitoring, implement conservative treatments, search for cause or Implement conservative treatments, search for cause, prepare for rapid delivery
4	Implement conservative treatments, search for cause, prepare for rapid delivery or Carry out rapid delivery, prepare for neonatal resuscitation
5	Carry out rapid delivery, prepare for neonatal resuscitation

#### Maternal PlGF level

**Fig. 2:**
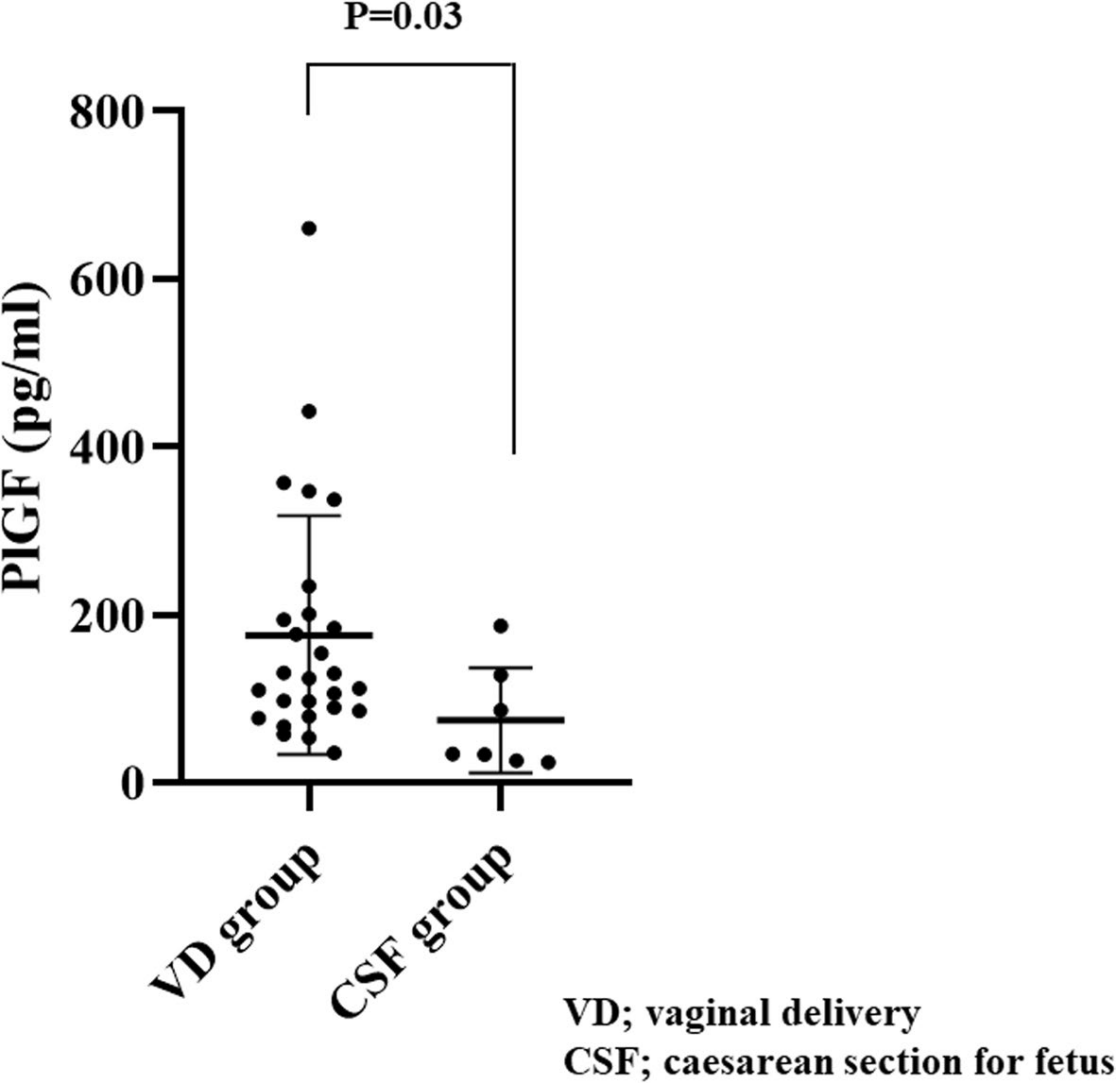
Level of placental growth factor (PlGF) in the vaginal delivery (VD) and cesarian section for fetal indications (CSF) groups

We compared the maternal PlGF level between the VD and CSF groups (Fig. 2). Prepartum PlGF level was significantly higher in the VD group (157 ± 106 pg/ml) than in the CSF group (74 ± 62 pg/ml) (*P* = 0.03). Hazard ratio of prepartum PlGF > 100 pg/ml was significantly higher (Hazard ratio 4.63; 95% Cl 1.12–23.5). Hazard ratio of age, gestational weeks, primipara, height, weight were not significant.

### Correlation between birth weight, pH of umbilical artery and PlGF

We analyzed the correlations between birth weight, umbilical artery pH, and PlGF. We found a positive but not significant correlation between birth weight and PlGF (*r *= 0.31, *P* = 0.07). Additionally, no significant correlation was found between umbilical artery pH and PlGF (*r *= 0.20, *P* = 0.26).

### Correlation between PlGF level and FHR monitoring parameters

**Fig. 3:**
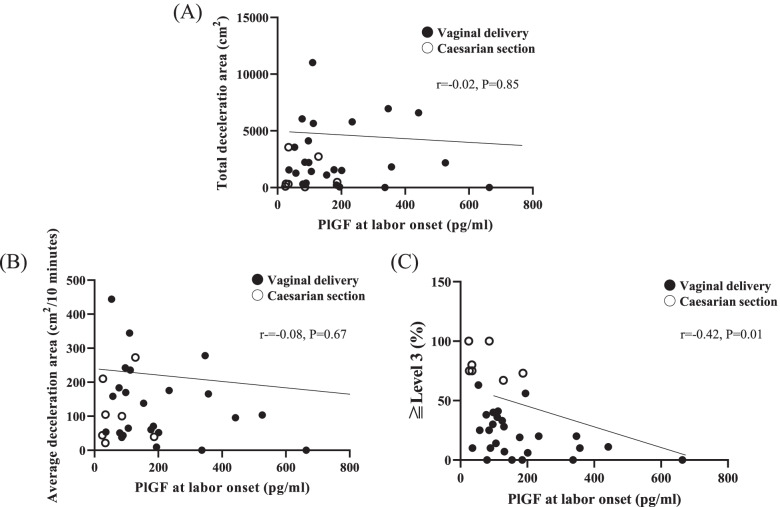
Relationship between placental growth factor (PlGF) and fetal heart rate monitoring parameters. (A) Total deceleration area, (B) average deceleration area, and (C) percentage of patients with level 3 or more in the five-tier classification system

We investigated the correlations between prepartum PlGF level and total deceleration area, average deceleration area, and the proportion of five-tier classification level 3 or higher (Fig. 3). There were no significant correlations between PlGF and total (*r *= -0.02, *P* = 0.85) or average (*r *= -0.08, *P* = 0.67) deceleration area (Fig. 2A and B). However, a significant negative correlation was observed between PlGF level and the proportion of five-tier classification level 3 or higher (*r *= -0.42, *P* = 0.01) as showed in Fig. 3C.

## Discussion

This study presented two main findings. First, PlGF might be a useful marker for predicting delivery outcomes. Second, PlGF correlated with FHR monitoring findings (five-tier classification level). The new finding of the present study is that PlGF correlated with one of two detailed FHR monitoring tools.

It is consistent with past findings that PlGF may be a useful marker for predicting delivery outcomes [12–17]. In these previous studies, the reasons why PlGF is associated with delivery outcome in normal pregnant women have not been addressed. PlGF is secreted from trophoblasts of placental villi [11, 18, 19], and its secretion decreases in the presence of hypoxic stress [11, 18, 19]. Moreover, in pregnant women without preeclampsia, the decrease in PlGF production is due to impaired placental function (impaired diffusion) in no decrease in PlGF caused by the excessive increase in sFlt-1. We speculate that this may be due to a decrease in PlGF production from the placenta caused by impaired placental function (impaired diffusion). This may be the reason why PlGF was more correlated with abnormal findings in placental function [11] and FHR monitoring during delivery. However, at present, there is insufficient evidence to make a clinical judgment based on PlGF alone, and clinical judgment should be made in combination with FHR monitoring and ultrasonography. That being said, PlGF is modified by various factors [20]. For example, in gestational diabetes, it increases owing to a compensatory angiogenesis mechanism in response to placental hypoxia induced by hyperglycemia [21]. Therefore, in this study, we excluded cases of gestational diabetes. In addition, because primiparous women have been reported to have low PlGF levels [10, 17], it is necessary to be mindful when using PlGF as a marker.

FHR can be monitored using various methods. A three-stage categorization for FHR monitoring patterns and their corresponding responses and management has been adopted by the American College of Obstetricians and Gynecologists, the Royal College of Obstetricians and Gynaecologists, and the Society of Obstetrics and Gynaecology of Canada. Later, in 2007, Parer and Ikeda proposed a five-tier classification, which has been adopted in Japan [22]. This five-tier classification system has not been adopted overseas because of its complexity and lack of evidence for its usefulness. However, since 2008, the five-tear classification has been reviewed as more useful than the three-stage classification for assessing the risk of fetal acidosis because it is more detailed than the three-stage classification [23–27]. Deceleration area has been reported to correlate with fetal acidosis, but this was not observed in the present study.

This study has some limitations. First, the sample size might have been too small for analysis. This occurred mainly because it was difficult to get blood samples after the onset of labor. Second, potential confounding factors that might affect PlGF synthesis and expression, such as the number of pregnancies and maternal age, remain unknown. In this context, it is important to note that we did not include cases that might have had extremely low PlGF, such as cases of fetal growth restriction, or extremely high PlGF, such as cases of diabetes mellitus. Nevertheless, birth weight tended to be lower in the CSF group.

While this study has several limitations, we were able to corroborate the relationship between PlGF level and fetal function by observing a correlation with FHR monitoring findings. Assessing risks to the fetus before delivery could be useful for the management of labor. Furthermore, it could help avoid emergency cesarean sections due to deteriorating fetal status. We believe that PlGF, in combination with other biomarkers or ultrasonography, could be a promising biomarker of fetal function. Further research is needed to confirm the usefulness of maternal PlGF as a predictive marker for delivery outcomes.

## Conclusion

PlGF measured before labor may be useful in predicting cesarean section due to fetal dysfunction. In addition, PlGF has a significant correlation with FHR findings at delivery by five-tier classification system.

## Data Availability

The data that support the findings of this study are available from the corresponding author, upon reasonable request.
